# Safety and effectiveness of a novel generator algorithm for bipolar vessel sealing: a randomised controlled chronic animal study

**DOI:** 10.1186/s12893-019-0625-2

**Published:** 2019-11-05

**Authors:** Bernhard Kraemer, Christos Tsaousidis, Stephan Kruck, Martin Schenk, Marcus Scharpf, Stefan Kommoss, Sara Brucker, Daniela Nuessle, Markus D. Enderle, Ulrich Biber

**Affiliations:** 1 University Hospital Tuebingen, Tuebingen, Germany; 2 Helios Klinikum Pforzheim, Pforzheim, Germany; 3 Erbe Elektromedizin GmbH, Waldhoernlestrasse 17, 72072 Tuebingen, Germany

**Keywords:** Swine, Animal model, Burst pressure, Thermal damage, Sealing time, Algorithm

## Abstract

**Background:**

Electrosurgical vessel sealers are gradually replacing conventional techniques such as ligation and clipping. Algorithms that control electrosurgical units (ESU), known as modes, are important for applications in different surgical disciplines. This chronic porcine animal study aimed to evaluate the safety and effectiveness of the novel thermoSEAL electrosurgical vessel sealing mode (TSM). The BiClamp® mode (BCM) of the renowned VIO® 300 D ESU served as control. BCM has been widely available since 2002 and has since been successfully used in many surgical disciplines. The TSM, for the novel VIO® 3 ESU, was developed to reduce sealing time and/or thermal lateral spread adjacent to the seal while maintaining clinical success rates. The primary aim of this study was to investigate the long-term and intraoperative seal quality of TSM.

**Methods:**

The BiCision® device was used for vessel sealing with TSM and BCM in ten German Landrace pigs which underwent splenectomy and unilateral nephrectomy during the first intervention of the study. The seals were cut with the BiCision® knife. Ninety-nine arteries, veins and vascular bundles were chronically sealed for 5 or 21 days. Thereafter, during the second and terminal intervention of the study, 97 additional arteries and veins were sealed. The carotid arteries were used for histological evaluation of thermal spread.

**Results:**

After each survival period, no long-term complications occurred with either mode. The intraoperative seal failure rates, i.e. vessel leaking or residual blood flow after the first sealing activation, were 2% with TSM versus 6% with BCM (*p* = 0.28). The sealing time was significantly shorter with TSM (3.5 ± 0.69 s vs. 7.3 ± 1.3 s, *p* < 0.0001). The thermal spread and burst pressure of arteries sealed with both modes were similar (*p* = 0.18 and *p* = 0.61) and corresponded to the histological evaluation. The measured tissue sticking parameter was rare with both modes (*p* = 0.33). Tissue charring did not occur. Regarding the cut quality, 97% of the seals were severed in the first and 3% in the second attempt (both with TSM and BCM).

**Conclusions:**

The novel TSM seals blood vessels twice as fast as the BCM while maintaining excellent tissue effect and clinical success rates.

**Trial registration:**

Not applicable.

## Background

Bipolar electrosurgical devices are becoming increasingly important for vessel sealing. These devices are gradually replacing conventional techniques such as ligation and clipping, especially in laparoscopic surgery. In addition to pure vessel sealers, there are also devices that not only seal vessels up to a diameter of 7 mm, but can also cut through the coagulated tissue [1–3], e. g. BiCision®, EnSeal® or LigaSure®. Besides geometry, materials and design of the devices where tissue contact occurs, the modes, i.e. tailor-made ‘ESU algorithms’, are particularly important for the respective application. In order to achieve an optimal interaction between ESU, device and tissue, special modes were developed and optimized with regard to the electrical parameters. One such new tissue-sparing mode for vessel sealing called “thermoSEAL mode” (TSM) was specially developed for the novel VIO® 3 ESU (Erbe Elektromedizin GmbH, Tuebingen, Germany). The mode permanently monitors the electrical tissue properties and offers an optimized setting for sealing different tissue types. Ideally, the sealing time and thermal lateral spread (TL) adjacent to the site of electrothermal fusion should be reduced compared to the clinically established pulsed BCM (automatically modulated sine-wave signal form with a fundamental frequency of 350 kHz as previously described [4]) of the well-known VIO® 300 D ESU, which has been successfully used for many years [4–6]. Previous ex vivo laboratory tests, in which post mortem tissue of explanted porcine renal arteries was sealed with TSM and BCM, as well as first in vivo porcine animal tests show that the TSM should be safe and effective in this sense [7].

A wide range of citations in peer reviewed scientific literature shows that the porcine model is established for preclinical studies during the development of thermo-surgical devices [2, 8–24]. The primary aim of this study was the safety evaluation of the TSM in terms of survival, long-term seal quality and intraoperative seal failure rate in a survival porcine animal model. The secondary aim was to evaluate the effectiveness associated with the use of the TSM and thus burst pressure (BP), TL, sealing time, tissue sticking to the device and charring of tissue were assessed.

## Methods

### Experimental animals

This study was reviewed and approved by the institutional review board for animal experiments, Regierungspraesidium Tuebingen, Germany (Approval No. F2/15) in compliance with the Animals Scientific Procedures Act 1986. Ten female German Landrace pigs aged approximately 12 weeks with a mean body weight of 41.3 ± 4.83 kg were used. The animals were obtained from a commercial pig breeder in Baden-Wuerttemberg, Germany and kept on straw in cages of 6.24 m^2^ (1–3 animals per cage) at a temperature of approximately 20 °C and a relative humidity of approximately 60% with natural illumination and tap water ad libitum. After a 7-day adaptation period, the pigs were starved in the morning before the surgical intervention. During the 5-day and 21-day survival period, the animals were housed as described above and clinically examined at least once a day by the veterinarian. All deviations from the expected course (e. g. refusal to eat, reduced mobility) were recorded, discussed and treated. In addition, the animals were evaluated by a scoring system that assessed the sensory awareness, food and water intake (appetence) and mobility behaviour.

### Premedication and anaesthesia

Before each surgical intervention, intramuscular premedication was performed using atropine (0.05 mg/kg, Dr. Franz Koehler Chemie GmbH Bensheim, Germany), azaperone (4.0 mg/kg, Elanco Animal Health, Bad Homburg, Germany), ketamine (14.0 mg/kg, Serumwerke Bernburg AG, Germany) and midazolam (1.0 mg/kg, ratiopharm GmbH, Ulm, Germany). A bolus of 2.0–5.0 mg/kg propofol (Fresenius Kabi, Bad Homburg, Germany) was administered intravenously prior to tracheal intubation. During the operation the animals were monitored, ventilated and kept in deep anaesthesia with 1.8% isoflurane (CP-Pharma Handelsgesellschaft mbH, Burgdorf, Germany) and fentanyl (ratiopharm GmbH, Ulm, Germany) at an intravenous dose of 30–100 μg/kg/h. Finally, the animals were euthanized with an intravenous injection of T 61 (Intervet Deutschland GmbH, Germany).

### Surgical procedure

Experienced surgeons (two gynaecologists and one urologist) with corresponding expertise in animal tests performed the surgical interventions. In the primary experiment (first intervention of the study), a midline laparotomy was performed, followed by splenectomy and unilateral nephrectomy according to the randomization (left or right). Incisions were made in both groins to locate the saphenous vessels. Splenic and renal arteries, veins and vascular bundles (combination of arteries and/or veins and connective tissue) as well as both saphenous arteries were compressed with a calibrated force and sealed with the BiCision® device. The Autostop function ended the activation. To aid location after nephrectomy and splenectomy and on second look, the seals were marked distally with fine sutures immediately after sealing without affecting the proximal sealing zone. The seals were severed with the cut function (knife) of the BiCision® with the exception of the saphenous arteries. All seals were thoroughly checked for leakage by macroscopic inspection before the abdomen and groin were closed. In the terminal experiment (second and terminal intervention of the study), after a survival period of either 5 or 21 days (5 animals per survival period), relaparotomy was performed and further blood vessels were sealed: the remaining contralateral renal arteries and veins, one of the carotid arteries, both axillary and femoral arteries and both jugular veins. After 2 h of prolonged survival period, these vessels were excised. Finally, the pigs were euthanized.

### Experimental design

This was a controlled, randomised, prospective and chronic animal study stratified by survival period (5 or 21 days). Five animals were used per survival period. The number of animals was selected to comply with FDA guidance “*Premarket Notification (510(k)) Submissions for Bipolar Electrosurgical Vessel Sealers for General Surgery*”. The bipolar vessel sealer BiCision® was used in combination with each of the following modes: in the test group blood vessels were sealed with the novel TSM, while in the control group the BCM was used. The blood vessels sealed in each animal were randomised to the test and the control group to minimise subjective bias. The TSM was operated by a VIO® 3 ESU (Erbe Elektromedizin GmbH, Tuebingen, Germany) with the setting Effect 1. The BCM was driven by a VIO® 300 D ESU (Erbe Elektromedizin GmbH, Tuebingen, Germany) with Effect 2. Both were the default setting for the BiCision® device of the respective ESU. At least ten vessels per mode and survival period should be sealed chronically for the evaluation of long-term seal quality. An estimated 45 arteries and veins per mode should be sealed in the terminal experiments and used for the ex vivo evaluation of TL and BP. The carotid arteries were sent in for histological evaluation of TL.

### Vessel diameter

The vessel diameter was determined in situ immediately before each sealing procedure using a calliper gauge and documented in both the primary and terminal experiments.

### Survival rate, long-term seal quality and intraoperative seal failure (primary aim)

A good state of health with positive weight development, as monitored by the veterinarian during the survival period, was documented as successful survival. Long-term seal quality was documented for vessels sealed during the primary experiment and was evaluated during the terminal experiment, including signs of complications or signs of bleeding or damage to the sealing site or to surrounding tissue macroscopically assessed for adjacent blood clots or minor hematomas. Intraoperative seal failure was documented in both the primary and terminal experiments. The definition of intraoperative seal failure was that the surgeon detected vessel leaking or residual blood flow immediately after the first sealing activation. In the event of such a failure, a maximum of two further activations were made to electrothermally seal the vessel. If this was *not* successful, the vessel was sutured.

### Sealing time

The sealing time (a secondary aim) was documented in both the primary and terminal experiments.

### Thermal lateral spread (TL) and burst pressure (BP)

The TL (a secondary aim) of the blood vessel specimens was obtained microscopically ex vivo by measuring the extent of the coagulation necrosis from the edge of the seal to the edge of the whitish discoloration of the tissue by coagulation. The sealed section of each vessel was then severed and both vascular stumps were subjected to ex vivo BP testing (a secondary aim). Physiological saline solution was instilled using a tailor-made, semi-automated BP test setup with continuous pressure monitoring. The pressure was increased until the fusion area leaked or burst. The maximum pressure was recorded as the BP value for each vascular stump. TL and BP were only documented for tissue samples from the terminal experiments (2 h survival period).

### Tissue sticking, tissue charring and cut quality

The tissue sticking to the device and charring of coagulated tissue (both secondary aims) were evaluated by the surgeon after each sealing procedure: 0 = “no sticking”, 1 = “sticking, but easy to remove the device”, 2 = “sticking and difficult to remove the device”, 3 = “sticking and difficult to remove the device and seal damaged” and 0 = “no charring”, 1 = “moderate charring”, 2 = “intense charring”. The cut quality was derived from the number of cut attempts per seal. These parameters were documented in both the primary and terminal experiments.

### Histological evaluation

During the terminal experiments, sealed carotid arteries with a 2-h survival period were excised, pinned on cork and fixed in 4.5% phosphate buffered formalin. They were cut longitudinally, embedded in paraffin and cut into 3 μm sections which were stained with haematoxylin and eosin (HE), Masson’s trichrome (MTC) and Picro Sirius Red (PSR) stain. The HE stain was used to obtain a histological overview. The MTC stain is a special staining technique that distinguishes the muscle from connective tissue and is particularly advantageous because the thermal damage zone with denatured protein/collagen has a bright red colour. For confirmation, PSR was additionally performed and viewed under the polarizing microscope. The PSR stain is intended for use in the histological visualization of collagen and muscle fibres in tissue sections. It is viewed using standard light microscopy or polarized light, which results in birefringence of intact collagen fibres and denatured tissue appears as non-birefringent.

### Statistical analysis

The statistical evaluation was done with GraphPad Prism (Version 7.01, U.S.A.). Since no considerable differences were observed between the group of all ten animals and the two groups of five animals per survival period, only the group of all ten animals was considered in the results section. Descriptive statistics (mean and standard deviation) were performed to describe the basic characteristics of the collected data. Fisher’s exact test was implemented to determine differences between two groups of categorical data (intraoperative seal failure, tissue sticking and charring) and the chi-squared test to determine differences between more than two groups. Differences between two groups of independent quantitative data (vessel diameter, sealing time, TL, BP, cut quality) were detected using a unpaired, two-tailed Student’s T-test provided the data were normally distributed, as confirmed by a Kolmogorov-Smirnov-Test. Otherwise, the Mann–Whitney test was performed. The equality of variance between two groups was checked with the F-test. Differences between independent quantitative data of more than two groups were determined by a one-way analysis of variance (ANOVA), provided that the data was normally distributed. If non-normal distribution was found for one of the groups, a Kruskal-Wallis test was used. *P*-values < 0.05 were considered significant.

## Results

### Vessel diameter

A total of 196 seals were performed during the primary and the terminal experiments, including 112 arteries, 66 veins and 18 vascular bundles. The mean vessel diameters did *not* differ between the two modes (*p* > 0.05), which was also true for certain vessel types (Table 1). In the primary experiment, 99 vessel seals (TSM: 50, BCM: 49) were performed. Among them were 48 arteries (TSM: 25, BCM: 23), 33 veins (TSM: 19, BCM: 14) and 18 vascular bundles (TSM: 6, BCM: 12). The mean vessel diameters did *not* differ between the two modes (Table 2). In the terminal experiment, 97 vessel seals (TSM: 48, BCM: 49) were performed. Among them were 64 arteries (TSM: 31, BCM: 33) and 33 veins (TSM: 17, BCM: 16). The mean vessel diameters did *not* differ between the two modes (Table 3).

**Table 1:** In situ vessel or vascular bundle diameters immediately before sealing (All vessels)

Vessel type	thermoSEAL mode (TSM)		BiClamp® mode (BCM)		*p* value
	No.	mean ± STD (range) [mm]	No.	mean ± STD (range) [mm]	
All vessels	98	4.4 ± 1.4 (2.1–9.6)	98	4.3 ± 1.4 (2.0–11)	0.65
Arteries	56	4.0 ± 0.9 (2.1–6.0)	56	4.1 ± 1.0 (2.5–6.8)	0.83
Veins	36	5.0 ± 1.9 (2.1–9.6)	30	4.7 ± 1.9 (2.0–11)	0.56
Renal arteries^a^	17	3.8 ± 0.9 (2.1–5.0)	15	4.3 ± 1.1 (3.0–6.1)	0.17
Femoral arteries	10	4.1 ± 0.8 (2.9–5.0)	10	4.0 ± 0.6 (3.5–5.4)	0.92
Axillary arteries	10	3.9 ± 0.6 (3.0–5.0)	10	3.5 ± 0.7 (2.5–4.3)	0.28
Renal veins	15	6.1 ± 1.8 (3.5–9.6)	16	5.5 ± 1.9 (3.7–11)	0.19
Jugular veins	9	2.9 ± 0.8 (2.1–4.2)	8	2.8 ± 0.5 (2.0–3.4)	0.76
Vascular bundles	6	4.7 ± 0.5 (4.0–5.5)	12	4.6 ± 1.8 (2.8–9.0)	0.89

^a^Cranial and caudal portions were often sealed separately, resulting in > 20 renal arteries (10 animals × 2 kidneys)

**Table 2:** In situ vessel or vascular bundle diameters immediately before sealing (Primary experiment)

Vessel type	thermoSEAL mode (TSM)		BiClamp® mode (BCM)		*p* value
	No.	mean ± STD (range) [mm]	No.	mean ± STD (range) [mm]	
All vessels	50	4.5 ± 1.5 (2.1–9.6)	49	4.4 ± 1.4 (2.2–9.0)	0.75
Arteries	25	3.7 ± 0.8 (2.1–5.9)	23	4.0 ± 1.1 (2.9–6.8)	0.79
Veins	19	5.4 ± 1.7 (3.2–9.6)	14	5.0 ± 1.3 (2.2–6.9)	0.45
Vascular bundles	6	4.7 ± 0.5 (4.0–5.5)	12	4.6 ± 1.8 (2.8–9.0)	0.89

**Table 3:** In situ vessel diameters immediately before sealing (Terminal experiment)

Vessel type	thermoSEAL mode (TSM)		BiClamp® mode (BCM)		*p* value
	No.	mean ± STD (range) [mm]	No.	mean ± STD (range) [mm]	
All vessels	48	4.3 ± 1.4 (2.1–8.1)	49	4.2 ± 1.5 (2.0–11)	0.79
Arteries	31	4.2 ± 0.8 (2.9–6.0)	33	4.1 ± 0.8 (2.5–6.1)	0.97
Veins	17	4.5 ± 2.1 (2.1–8.1)	16	4.4 ± 2.3 (2.0–11)	0.95

### Survival rate

There were no adverse events and none of the animals (0/10) died after the primary experiment or during the survival periods. In all animals (10/10), seroma formation could be observed postoperatively at the surgical site in the groin area, which did *not* lead to any clinically relevant impairment. Apart from this, there were no postoperative anomalies. This was in line with the weight gain of the animals (10/10), which corresponded to the weight gain observed in healthy pigs fed on a needs-based, non-compulsory diet. Apart from postoperative analgesia and prophylactic antibiotics, no further veterinary interventions were necessary.

### Long-term seal quality

A total of 99 vessel sealings (TSM: 50, BCM: 49) with similar mean vessel diameters (TSM: 4.5 mm, BCM: 4.4 mm, Table 2) and diameter ranges (TSM: 2.1–9.6 mm, BCM: 2.2–9.0 mm, Table 2) for both modes were assessed for long-term seal quality. There was no difference between TSM and BCM, i.e. the clinical success rate was 100% for both modes. On relaparotomy after 5 days, the sealing sites and the surrounding tissue did *not* show any signs of complications or evidence of bleeding. The same is true for relaparotomy after 21 days with even more advanced or fully completed healing.

### Intraoperative seal failure

Intraoperative seal failures were rare (total: 8/196 ≈ 4%) and comparable between TSM and BCM (2/98 ≈ 2% vs. 6/98 ≈ 6%, *p* = 0.28). Of these eight failures, two arteries and two veins could be resealed with a maximum of two further sealing activations, as allowed per protocol. Three further vessels were sutured: a renal artery and a splenic vein as resealing was *not* possible because the remaining vascular stump was too short to be grasped with the vessel sealer and a femoral artery since the other two sealing activations were unsuccessful. One vascular bundle was *not* resealed because the seal failure only occurred on the side adjacent to an explanted spleen.

### Sealing time

**Fig. 1:**
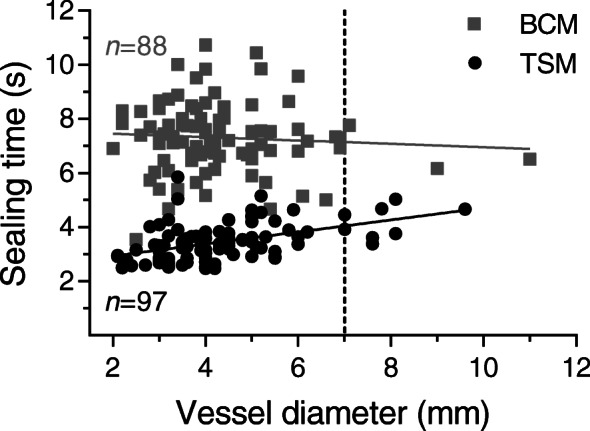
Sealing time vs. vessel diameter of sealed arteries, veins and vascular bundles for TSM (black circles) and BCM (grey squares). TSM showed a significant positive correlation (*p* < 0.0001, *R*^*2*^ = 0.21), whereas BCM did not (*p* = 0.57, *R*^*2*^ = 0.005). The vertical dashed line shows the maximum diameter approved with the BiCision® vessel sealer (7 mm). This figure was presented on a poster [25]

The sealing time of all vessels was significantly shorter for TSM (3.5 ± 0.7 s) compared to BCM (7.3 ± 1.3 s, *p* < 0.0001). The same is true for arteries, veins, renal arteries or vascular bundles (*p* < 0.0001, Table 4). The sealing time with the BCM did not correlate with vessel diameter, while with the TSM a significant positive correlation between these parameters with a slope of 0.22 s/mm was observed (Fig. 1).

**Table 4:** Sealing time (Primary and terminal experiment)

Vessel type	thermoSEAL mode (TSM)		BiClamp® mode (BCM)		*p* value
	No.	mean ± STD (range) [mm]	No.	mean ± STD (range) [mm]	
All vessels	97	3.5 ± 0.69 (2.5–5.9)	88	7.3 ± 1.3 (3.5–11)	< 0.0001
Arteries	55	3.4 ± 0.65 (2.5–5.9)	51	7.4 ± 1.4 (3.5–11)	< 0.0001
Veins	36	3.6 ± 0.78 (2.5–5.2)	27	7.4 ± 1.4 (4.7–10)	< 0.0001
Renal arteries	17	3.5 ± 0.45 (2.8–4.4)	15	7.4 ± 1.4 (5.1–9.8)	< 0.0001
Vascular bundles	6	3.7 ± 0.54 (3.0–4.4)	10	6.9 ± 0.87 (5.7–8.7)	< 0.0001

6% (11/196) of the vessels seals with unintended multiple activations could not be evaluated

### Thermal lateral spread (TL)

Seventy-four of 97 (≈76%) sealed blood vessels (52 arteries, 22 veins) from the terminal experiments were used to measure TL. Twenty-three blood vessels (≈24%) could *not* be assessed due to insufficient visibility of TL. This can be explained by the in vivo setting, which is associated with more blood on the specimens and possible rupture of the specimens. Overall, TL near the sealing sites (Table 5) was similar with TSM and BCM (*p* = 0.61) with an approximate mean extent of 0.7 mm. There was also no difference between the two modes regarding arteries (*p* = 0.96), veins (*p* = 0.28) or other anatomically specified vessel types (*p* > 0.05). A mode-independent comparison of the TL of the different blood vessel types also showed no significant differences (*p* = 0.65).

**Table 5:** Thermal lateral spread (TL)

Vessel type	thermoSEAL mode (TSM)		BiClamp® mode (BCM)		*p* value
	No.	mean ± STD (range) [mm]	No.	mean ± STD (range) [mm]	
All vessels	40	0.68 ± 0.32 (0–1.4)	34	0.72 ± 0.41 (0–1.4)	0.61
Arteries	27	0.67 ± 0.31 (0–1.2)	25	0.68 ± 0.45 (0–1.4)	0.96
Veins	13	0.69 ± 0.34 (0.2–1.4)	9	0.85 ± 0.27 (0.4–1.4)	0.28
Renal arteries	7	0.74 ± 0.13 (0.6–1.0)	6	0.70 ± 0.48 (0.2–1.4)	0.81
Femoral arteries	10	0.64 ± 0.43 (0–1.2)	8	0.49 ± 0.40 (0–1.0)	0.46
Axillary arteries	9	0.64 ± 0.28 (0.2–1.0)	10	0.85 ± 0.46 (0.1–1.4)	0.26
Renal veins	5	0.65 ± 0.28 (0.3–1.0)	4	1.0 ± 0.26 (0.7–1.4)	0.19
Jugular veins	8	0.72 ± 0.39 (0.2–1.4)	5	0.72 ± 0.24 (0.4–1.0)	0.99

24% (23/97) of the vessels could not be evaluated (see Results section TL)

### Burst pressure (BP)

**Fig. 2:**
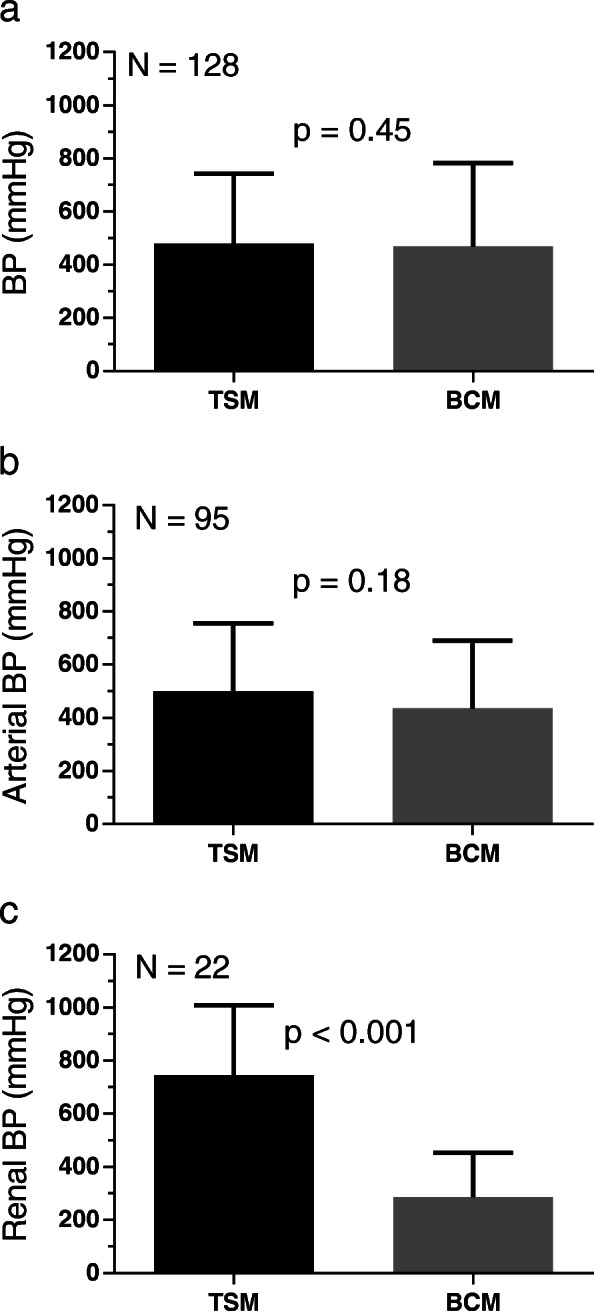
BP for TSM (black) and BCM (grey) with the BiCision® device: **a** Arteries and veins. *N* = 128. **b** Arteries. *N* = 95. **c** Renal arteries. *N* = 22. Figure b was presented on a poster [25]

The BP of 128 vascular stumps tested ex vivo was similar for TSM and BCM (479 ± 263 mmHg vs. 469 ± 313 mmHg, *p* = 0.45, Table 6 and Fig. 2a). These 128 stumps were taken from 90 transected vessels (TSM: 45, BCM: 45) corresponding to 180 vascular stumps. Since the sensitive tissue quality of some specimens did *not* allow a connection to the experimental BP test bench, 52 stumps (≈29%) could *not* be used in this test (TSM: 21 ≈ 23%, BCM: 31 ≈ 34%). Based on the BP values, it was decided to analyse subgroups of veins smaller or larger than 5 mm in diameter. In addition, the arteries were divided into groups according to their anatomical origin. There was no difference between the modes concerning arteries (*p* = 0.18, Fig. 2b), veins (*p* = 0.62), femoral arteries (*p* = 0.67), axillary arteries (*p* = 0.90), renal veins (*p* = 0.20) and veins with a diameter smaller than 5 mm (*p* = 0.32) or larger than 5 mm (*p* = 0.29). Renal arteries showed significantly higher BP values for the TSM (*p* < 0.001, Fig. 2c) and jugular veins showed significantly higher BP values for the BCM (*p* < 0.05). Regardless of the mode, the BP of axillary arteries was higher than that of the femoral arteries (507 ± 253 mmHg vs. 371 ± 179 mmHg, *p* = 0.01) and the BP of veins smaller than 5 mm was higher than that of veins larger than 5 mm (609 ± 360 mmHg vs. 182 ± 71.6 mmHg, *p* < 0.0001). A comparison of the BPs of renal and jugular veins showed higher values for the jugular veins with both TSM and BCM (*p* < 0.01).

**Table 6:** Ex vivo burst pressure (BP)

Vessel type	thermoSEAL mode (TSM)		BiClamp® mode (BCM)		*p* value
	No.	mean ± STD (range) [mm]	No.	mean ± STD (range) [mm]	
All vessels	69	479 ± 263 (57–1116)	59	469 ± 313 (72–1430)	0.45
Arteries	49	499 ± 255 (57–1102)	46	435 ± 255 (72–1090)	0.18
Veins	20	431 ± 282 (60–1116)	13	587 ± 457 (171–1430)	0.62
Renal arteries	12	745 ± 264 (57–1102)	10	286 ± 166 (72–606)	< 0.001
Femoral arteries	16	333 ± 113 (170–620)	15	411 ± 227 (115–876)	0.67
Axillary arteries	19	509 ± 221 (267–1050)	19	504 ± 287 (80–1090)	0.90
Renal veins	6	163 ± 72.9 (60–266)	6	210 ± 41.6 (171–273)	0.20
Jugular veins	14	545 ± 259 (300–1116)	7	910 ± 390 (360–1430)	0.019
Veins < 5 mm	15	522 ± 265 (198–1116)	9	743 ± 460 (188–1430)	0.32
Veins > 5 mm	5	156 ± 79.2 (60–266)	4	215 ± 51.8 (171–273)	0.29

The *n* given represents the number of vascular stumps used for BP testing. 29% (52/180) of the vascular stumps could not be evaluated (TSM: 21 ≈ 23%, BCM: 31 ≈ 34%) (see Results section BP)

### Tissue sticking, tissue charring and cut quality

Tissue sticking to the vessel sealer was relatively uncommon and similar for TSM and BCM (13/98 ≈ 13% vs. 19/98 ≈ 19%, *p* = 0.33). The occurrence of sealing events without sticking (rating 0) did *not* differ between TSM and BCM (85/98 ≈ 87% vs. 79/98 ≈ 81%, *p* = 0.33), which also applied to the other ratings (Table 7). Tissue charring was classified as non-existent regardless of the mode, i. e. 100% (98/98) of the vessels were rated ‘0’ in both cases. The cut quality was very similar for TSM and BCM (1.03 ± 0.16, *n* = 39 vs. 1.03 ± 0.16, *n* = 39), i.e. 97% (38/39) of the seals were cut in the first attempt and only 3% (1/39) were cut in the second attempt regardless of the mode.

**Table 7:** Rating of tissue sticking to the vessel sealer

Rating level	thermoSEAL mode (TSM)		BiClamp® mode (BCM)		*p* value
	No.	Percentage [%]	No.	Percentage [%]	
Rating 0	85/98	87	79/98	81	0.33
Rating 1	9/98	9	9/98	9	1.00
Rating 2	3/98	3	7/98	7	0.33
Rating 3	1/98	1	3/98	3	0.62

Ratings: 0 = no sticking, 1 = sticking, but easy to remove the device, 2 = sticking and difficult to remove the device, 3 = sticking and difficult to remove the device and seal damaged

### Histology

**Fig. 3:**
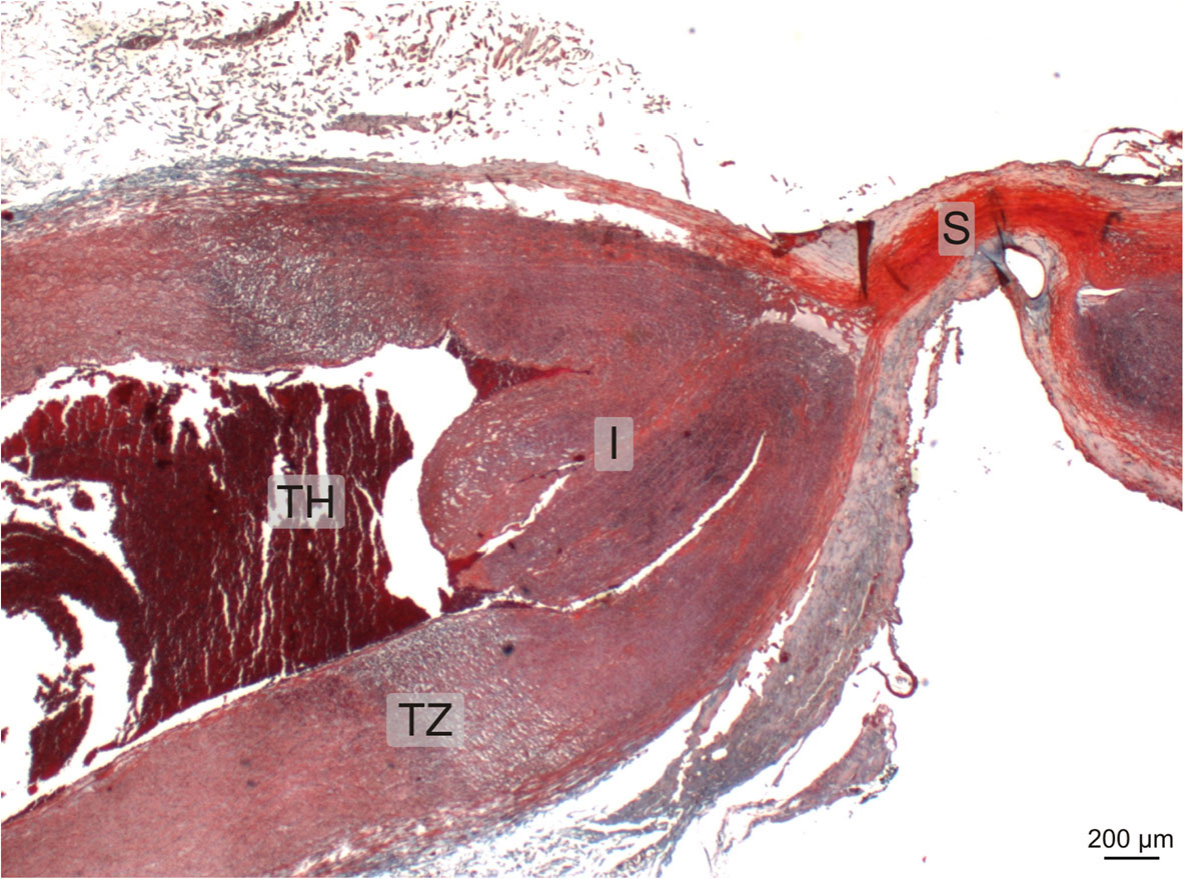
Histological section of a typical thermally sealed artery stained with MTC: The sealing site (S, upper right) shows the bright red colour of denatured collagen. The invagination of the media (I) and a thrombus (TH) can be seen. The transition zone (TZ) between denatured and unaffected arterial structures is characterised by a broad transmural zone of intra- and extracellular vacuolisation

**Fig. 4:**
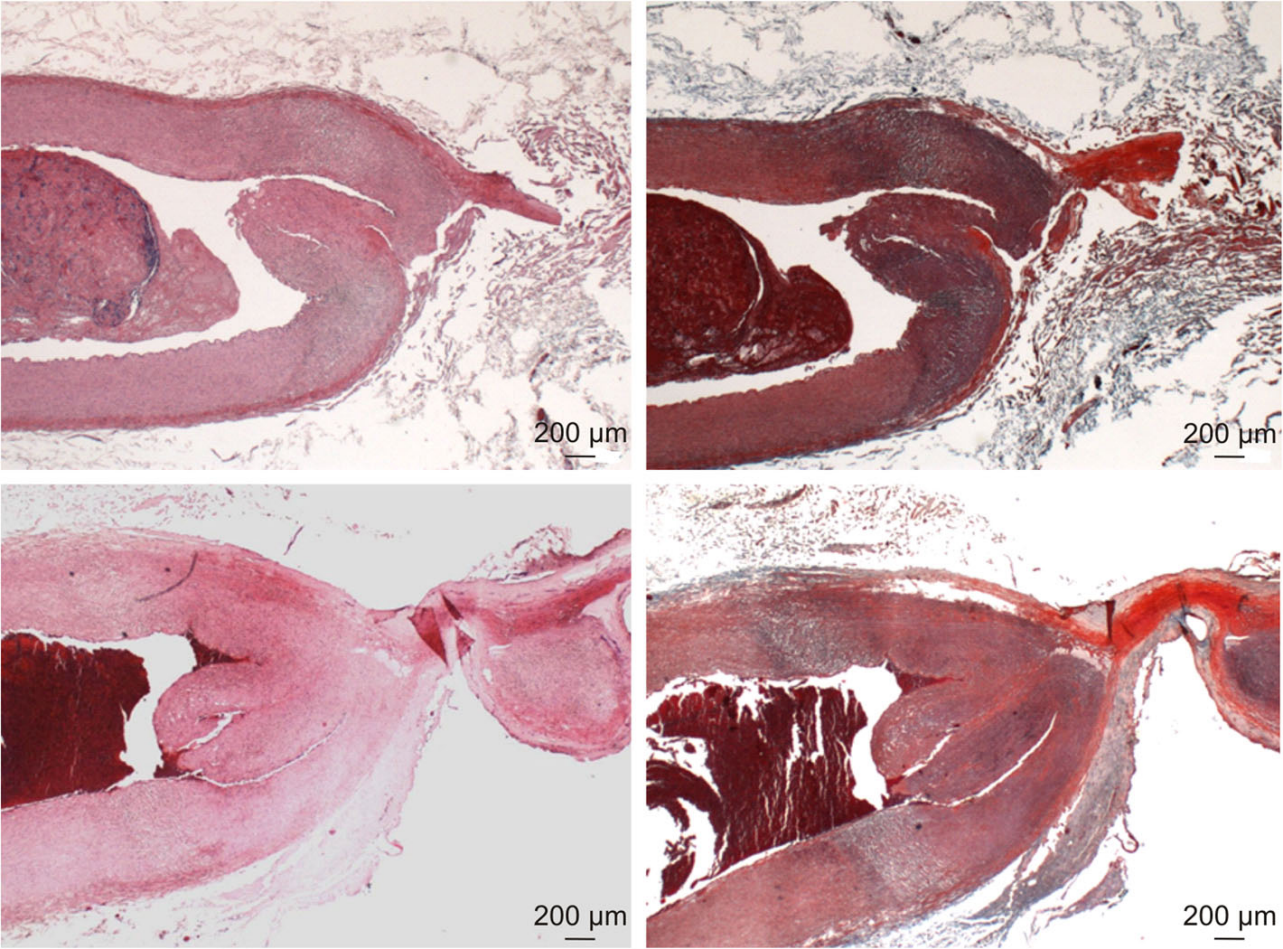
Histological comparison between two thermally sealed arteries (BCM top, TSM bottom, left HE stain, right MTC stain): Both samples show similar alterations caused by the sealing process: strong thermal alterations with denaturation of protein components in the vessel wall, of the adventitia and media next to the sealing sites and a transition zone between unaffected and affected media

Seven thermally sealed carotid arteries were examined at microscopic level (4 x BCM, 3 x TSM). All samples except one (BCM#3) showed fusion of the arterial walls with complete occlusion of the arterial lumen. The arterial walls directly adjacent to the sealing site showed transmural thermal alterations with strong denaturation in the outer third, affecting adventitia and media. The transition from normal arterial structures to arterial wall affected by surgery was characterized by a broad transmural zone of intra- and extracellular vacuolisation (Fig. 3). This zone was observed equally in both groups, as well as minor thrombus formation in the arterial lumen adjacent to the sealing site (Fig. 4). Most vessels (6/7 ≈ 86%) showed a complete invagination of the interrupted media into the arterial lumen, which is typical for electrothermal vessel sealing (Table 8).

**Table 8:** Histological properties of sealed carotid arteries

Property	Sample #						
	BCM#1	BCM#2	BCM#3	BCM#4	TSM#1	TSM#2	TSM#3
Occlusion of arterial lumen	Yes	Yes	No^a^	Yes	Yes	Yes	Yes
Transition zone	Broad	Broad	Small	Broad	Broad	Broad	Broad
Thrombus formation	Yes	No	Yes, strong	No	Yes	Yes	Yes
Invagination of the media	Complete	Complete	Incomplete	Complete	Complete	Complete	Complete

^a^The arterial lumen reopened, probably because of the mechanical excision of the specimen from the animal

## Discussion

Both TSM and BCM showed a low intraoperative seal failure rate (≈2% vs. ≈6%). Regardless of mode, seal failures in arteries, veins and vascular bundles were similarly rare (≈4%, ≈5% and ≈6%, *p* = 0.65). Clinically, this is a remarkable result. Richter et al. also observed no differences between arteries and veins with regard to seal failure [11]. It is known that seal failure rates increase with vessel diameter [26]. Although vessels with a large diameter range (2.0–11.0 mm) were sealed, this correlation could not be confirmed, probably due to the low seal failure rate, few vessels with a diameter above 7 mm (*n* = 9) and the resulting lack of statistical power. Therefore, no difference in vessel diameter was observed for failed seals compared to successful seals (4.7 ± 1.2 mm, *n* = 8 vs. 4.3 ± 1.4 mm, *n* = 188, *p* = 0.34). All ten animals survived 5 or 21 days and on second look the sealing sites and surrounding tissue showed no signs of complications or evidence of bleeding. The optimal long-term seal quality and the excellent intraoperative seal failure rate show that the primary aim was fulfilled. The validity of the results is confirmed by the fact that the composition of the vessels and their diameters were very similar for TSM and BCM (Tables 1, 2 and 3).

**Fig. 5:**
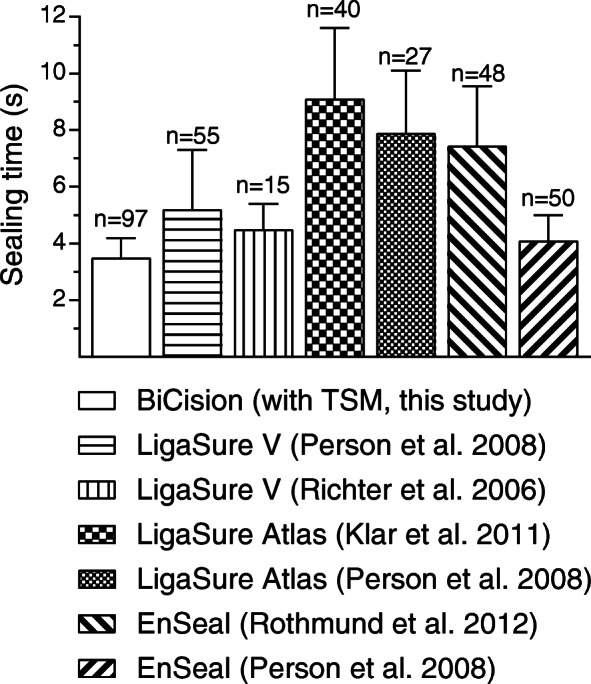
Sealing times of different electrosurgical vessel sealers. Data is included from this study (no fill pattern) and other in vivo animal studies (Klar et al. [27], Person et al. [13], Richter et al. [11], Rothmund et al. [28])

The mean sealing time of the TSM was 3.5 s and shorter than that of the BCM (*p* < 0.0001). This shortening of the sealing time with TSM was indicated by earlier in vivo animal tests [7] and was confirmed here with a higher number of samples. It is remarkable that a significant positive correlation between sealing time and vessel diameter was observed for the TSM, reflecting an appropriate function of the closed loop electrical regulation, taking into account the change in tissue impedance during the sealing process. In addition, the sealing time of the TSM varied only slightly and less than that of the BCM (S.D. = 0.69 s vs. S.D. = 1.3 s, *p* < 0.0001). Low variance in sealing time should have a positive effect on handling and the feeling of safety. Compared to other vessel sealers used in recent in vivo animal studies [11, 13, 27, 28], the sealing time of the TSM is relatively short (Fig. 5). Short sealing times are beneficial and lead to a shorter total surgery time, especially in laparoscopy. The aim of shortening the sealing time while maintaining clinical success rates was achieved by further developing the ESU’s regulation.

**Fig. 6:**
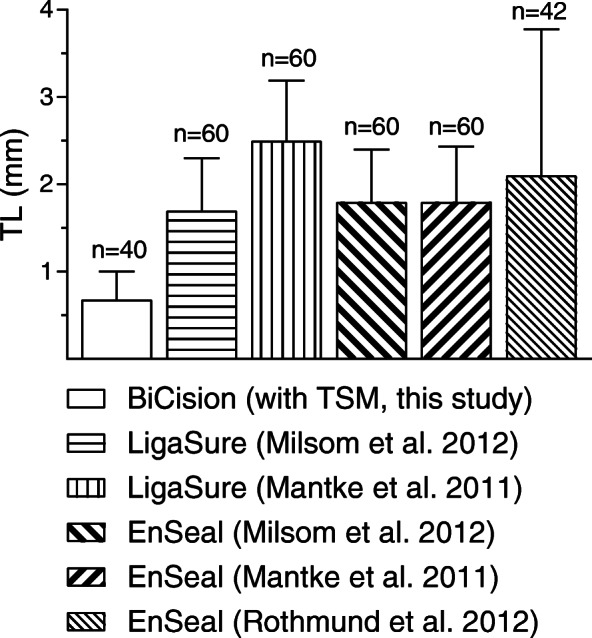
TL of different vessel sealers. Data is included from this study (no fill pattern) and other in vivo (Milsom et al. [20], Rothmund et al. [28]) and ex vivo (Mantke et al. [29]) animal studies

The TL was very similar with TSM and BCM (*p* = 0.61, Table 5). The extent of TL is competitive (Fig. 6) with other vessel sealers [20, 29]. The TL was independent of the vessel type (*p* = 0.65). This is in line with data from others [14, 20] and a previous report from us [28]. It was reported that TL increases with vessel diameter [20], but this correlation was not confirmed (*p* = 0.49, *R*^*2*^ = 0.01). Moreover, a significant reduction in TL with TSM compared to BCM as demonstrated by ex vivo bench tests [7] could *not* be confirmed here with a higher number of samples. Since this study showed no difference in TL and the ex vivo tests showed less TL with TSM, these results taken together suggest that TSM could cause even less, but definitely not more TL compared to BCM. The aim of reducing TL at consistent clinical success rates may have been achieved even though the standard with BCM is high.

**Fig. 7:**
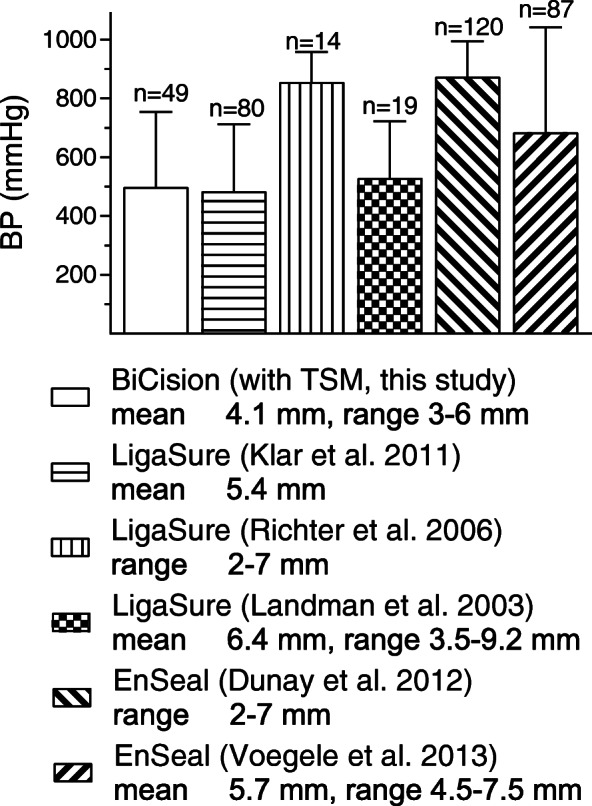
Arterial BP of this study (no fill pattern) in comparison to other in vivo (Klar et al. [27], Richter et al. [31], Landman et al. [9], Dunay et al. [18] [includes 30 veins and 30 arteries]) and ex vivo (Voegele et al. [32]) animal studies. Mean and/or range of the arterial vessel diameters in the legend. *n* is the number of vascular stumps

BP values were similar with TSM and BCM (Table 6). Bench testing with renal arteries showed a BP of 774 ± 190 mmHg for the TSM, which was similar in this study (Fig. 2c, 745 ± 264 mmHg). Arterial BPs show a dependence on anatomical origin so that renal arteries can withstand the highest pressure, followed by axillary arteries, and the lowest BPs were measured for femoral arteries. Sindram et al. also showed decreasing BPs for renal, carotid, iliac and femoral arteries, suggesting that collagen content is the most important determinant of BP [17]. The vessel diameter, which is also a predictor for BP, was *not* significantly different for these artery groups, as was the case for the arteries in this study. Latimer et al. also showed that collagen content is a good predictor for BP of human blood vessels with a diameter of < 5 mm using LigaSure Atlas, whereas it is less important for vessels with a diameter of > 5 mm [30]. This suggests that the variance in arterial BP can mainly be explained by collagen content, while the vessel diameter seems to be negligible up to at least 5 mm. A comparison of our BP values with other animal tests [9, 18, 27, 31, 32] using LigaSure or EnSeal is shown in Fig. 7. A statistical comparison of the values is *not* recommended as there are considerable differences in vessel diameter and composition. However, it can be concluded that an appropriate number of vessels were tested in this study and that the arterial BP values of the TSM are competitive. The mean venous BP of the TSM was 492 ± 363 mmHg. This comparatively high value can be explained by the different types of veins and their diameters. If only renal veins typically > 5 mm in diameter were considered (Table 1), the mean BP was 163 ± 72.9 mmHg. This value is consistent with a study by Landmann et al., who reported a mean BP of 233 mmHg for 11 porcine renal veins [9]. In contrast, we found that the jugular veins with a mean diameter of merely 2.9 ± 0.8 mm (Table 1) had BP values greater than 500 mmHg, which is untypically high for veins and higher than, for example, the values obtained in 2011 with EnSeal (262 ± 51 mmHg, *n* = 14) [33]. However, a number of studies report high BP values for certain veins: Dunay et al. obtained high BP values for jugular veins using EnSeal (lowest value 425 mmHg) [18]. Overhaus et al. reported a mean BP of 425 ± 74 mmHg for small porcine veins (diameter 1–5 mm) using EnSeal, which was even higher than in arteries of similar diameter [34]. Kennedy et al. reported a mean BP of 378 ± 211 mmHg for porcine renal, splenic, ovarian and uterine veins (diameter 1–7 mm, *n* = 13) using a prototypic bipolar vessel sealer [2]. Furthermore, Noble et al. obtained BP values above 1000 mmHg for ex vivo human veins with a diameter of ~ 1 mm using LigaSure and other devices [35]. When interpreting experimental BP results, it should be borne in mind that these are usually far above the physiological blood pressure values. In summary, it can be said that arterial and venous BP values measured for the two modes are competitive with and in agreement with other measurements presented in the current literature.

Electrosurgical devices are generally known to occasionally stick to tissue and tend to accumulate layers of contamination [28, 31]. This may be clinically relevant, particularly in laparoscopy, as forced movements to release the device from the coagulated and transected tissue may result in seal failure. Regardless of the mode, tissue sticking to the device was not common in this study (≈13% vs. ≈19%, *p* = 0.33, Table 7). Therefore, the novel TSM is neither a significant improvement nor deterioration in tissue sticking. Tissue charring was considered non-existent in both modes. Regarding the cut quality, 97% of the seals were cut in the first attempt and only 3% were cut in the second attempt with both modes. This corresponds to the previously obtained data, which show a very high cut quality of BiCision® [28]. Since the evaluation of tissue sticking, tissue charring and cut quality is subjective and user-dependent, the interpretation of the result of these aspects remains a methodological limitation.

The histological evaluation of the successfully sealed vessels showed no differences between the two modes. The distribution of thermal alterations and the invagination of the media typical for electrothermally sealed vessels were identical. One caveat is the small number of specimens evaluated. Nevertheless, the histological results agree with the macroscopic evaluation of TL, which also showed no differences between TSM and BCM.

## Conclusions

Apart from technical specifications of an electrosurgical device, especially the geometry of the device jaws which are in direct contact with the target tissue, the ESU’s algorithm plays an important role for fast, safe and successful vessel sealing. The further development of user-friendly intelligent modes is therefore just as important as the design of ergonomic instruments. In this context, our study presents the novel TSM, which seals blood vessels twice as fast as the BCM, while providing excellent tissue effect and clinical success rates.

## Data Availability

The data that support the findings of this study are available from Erbe Elektromedizin GmbH, but restrictions apply to the availability of these data, which were used under license for the current study, and so are not publicly available. Data are however available from the authors upon reasonable request and with permission of Erbe Elektromedizin GmbH.

## Abbreviations

BCM: BiClamp® mode
BP: Burst pressure
ESU: Electrosurgical unit
HE: Haematoxylin and eosin
MTC: Masson’s trichrome
PSR: Picro Sirius Red
TL: Thermal lateral spread
TSM: ThermoSEAL mode
